# Renal denervation: are we at a crossroads?

**DOI:** 10.1007/s12471-016-0856-0

**Published:** 2016-06-09

**Authors:** M. Voskuil

**Affiliations:** Department of Cardiology, University Medical Center Utrecht, Heidelberglaan 100, 3584 CX Utrecht, The Netherlands

Since the first publication by Henry Krum in 2009 [1], it has been a bumpy road for renal denervation (RDN) for the treatment of sympathetic hyperactivity associated disease states, such as hypertension. After initial positive results of non-randomised and randomised trials, a true hype arose with more than 50 companies developing their own RDN system. The procedure was reimbursed in several countries in Europe and within a few years 15,000 to 20,000 procedures were performed. However, in 2014, the presentation of the sham-controlled blinded Symplicity HTN-3 trial at the scientific sessions of the American College of Cardiology and simultaneous publication in the New England Journal of Medicine caused a 180° turn in the thinking of many physicians about this potential novel treatment modality [2]. In contrast with previous trials, in Symplicity HTN-3 the mean decrease in office systolic blood pressure was only 14.13 mmHg in the treated group, but more surprisingly it was 11.74 mmHg in the sham control group. This resulted in a modest 2.39 mmHg difference in the RDN treated versus control group, lower than the preset superiority margin of 5 mmHg and thus not statistically significant. The differences observed using ambulatory blood pressure measurements (ABPM) were also too small to reach statistical significance (‑6.75 mmHg and -4.79 mmHg, respectively; a difference of -1.96 mmHg). The aftermath of these unexpected results caused subsequent trials to directly suspend patient recruitment. Furthermore, in most countries attention for RDN studies and referrals of patients declined steeply. Several important issues were identified such as the small number of patients with resistant hypertension being found to be eligible for RDN [3]. Also, blood pressure responses to RDN using the commercially available catheters were found highly variable, with a considerable number of non-responders. Third, the absence of good predictors and a periprocedural read-out of response to RDN is seen as a big caveat. Searching for an explanation for the neutral results of the Symplicity HTN-3 trial, among others the efficacy of the used catheter was questioned. In this light, besides renal nerve ablation with radiofrequency energy using different catheters, several other techniques came onto the market using alcohol injection and high frequency ultrasound. Also, a system to deliver energy to renal nerves using low-intensity focused ultrasound through an external ultrasound source has been developed and is currently being tested in a first-in-man study.

Alternatively, Prochnau and colleagues used a standard radiofrequency ablation catheter in their study in the current issue of the Netherlands Heart Journal [4]. For this study the authors used 24-hour ABPM instead of the less reliable office measurements, both for screening and follow-up of the patients. A theoretical advantage is the steerability of the amount of energy used and in general the great experience with the described catheter for standard electrophysiology procedures. Using this catheter it was possible to induce a larger amount of energy (up to 12 watts in their hands), potentially inducing deeper penetrating ablations. Unfortunately, the authors do not describe the strategy and/or mean amount of energy used (range 8‑12 watts). Using this technique they were able to reduce ABPM -15/-7 ± 18/13 mmHg at 12 months of follow-up. Even after correction for multiple testing this was a statistically significant change compared with the baseline measurements. However, it must be stated that bias was introduced by excluding 10 (out of 70) patients who did not respond to the treatment and were referred for alternative RDN techniques. Although patients were followed in a stringent way using ABPM, no firm conclusions can be made on the results of the current analysis, other than that RDN using this electrophysiology catheter is safe. The main limitation of the study is the lack of a control group. We know from contemporary trials in hypertension that in placebo arms a mean systolic blood pressure fall of 8.76 mmHg is observed [5]. Taking this placebo effect into account and including the data of the non-responders, the results of the current analysis may well have been neutral.

In general, after an initial phase of testing new treatment techniques for safety in phase 1‑2 trials, the discussed data stress the need for phase 3 studies in the field of hypertension, using only a randomised sham-controlled design.

Also, post-hoc analyses from the Symplicity HTN-3 trial show that patient selection is the key in studying the efficacy of RDN in the treatment of hypertension. These studies suggest that blood pressure reduction after RDN might be influenced by the presence of obstructive sleep apnoea and racial differences and even seems to be time-dependent [6–8]. Although these analyses are no more than hypothesis generating, Simplicity HTN-3 has taught us some lessons for upcoming trial designs.

Future research in renal denervation is likely to focus on three main areas: improved catheter design/alternative techniques for effective denervation, periprocedural assessment of the efficacy of denervation, and identification of the subgroups most likely to benefit from treatment. The currently running Spyral HTN OFF-MED and Spyral HTN ON-MED using a multiple electrode catheter will hopefully shed some light in this era of uncertainty [9]. Otherwise, going from the bedside back to the bench might be necessary to get this interesting field of research forward.
